# Carbohydrate Mimetic Peptides Augment Carbohydrate-Reactive Immune Responses in the Absence of Immune Pathology

**DOI:** 10.3390/cancers3044151

**Published:** 2011-11-11

**Authors:** Leah Hennings, Cecile Artaud, Fariba Jousheghany, Behjatolah Monzavi-Karbassi, Anastas Pashov, Thomas Kieber-Emmons

**Affiliations:** Winthrop P. Rockefeller Cancer Institute and Department of Pathology, University of Arkansas for Medical Sciences, Little Rock, AR 72205, USA; E-Mails: LHennings@uams.edu (L.H.); cecile.artaud@pasteur.fr (C.A.); JousheghanyFariba@uams.edu (F.J.); KarbassiBehjatolahM@uams.edu (B.M.-K.); PashovAnastas@uams.edu (A.P.)

**Keywords:** antibodies, vaccination, cancer, carbohydrate, glycans, peptide

## Abstract

Among the most challenging of clinical targets for cancer immunotherapy are Tumor Associated Carbohydrate Antigens (TACAs). To augment immune responses to TACA we are developing carbohydrate mimetic peptides (CMPs) that are sufficiently potent to activate broad-spectrum anti-tumor reactivity. However, the activation of immune responses against terminal mono- and disaccharide constituents of TACA raises concerns regarding the balance between “tumor destruction” and “tissue damage”, as mono- and disaccharides are also expressed on normal tissue. To support the development of CMPs for clinical trial testing, we demonstrate in preclinical safety assessment studies in mice that vaccination with CMPs can enhance responses to TACAs without mediating tissue damage to normal cells expressing TACA. BALB/c mice were immunized with CMPs that mimic TACAs reactive with *Griffonia simplicifolia* lectin 1 (GS-I), and tissue reactivity of serum antibodies were compared with the tissue staining profile of GS-I. Tissues from CMP immunized mice were analyzed using hematoxylin and eosin stain, and Luxol-fast blue staining for myelination. Western blots of membranes from murine mammary 4T1 cells, syngeneic with BALB/c mice, were also compared using GS-I, immunized serum antibodies, and naive serum antibodies. CMP immunization enhanced glycan reactivities with no evidence of pathological autoimmunity in any immunized mice demonstrating that tissue damage is not an inevitable consequence of TACA reactive responses.

## 1. Introduction

Anti-tumor immune responses are a facet of the tissue-specific autoimmune phenomenon [1], therefore the generation of immune responses to tissue rejection antigens represents an important conceptual approach in cancer immunotherapy. Tumor-associated carbohydrate antigens (TACAs) are potential tissue rejection antigen targets that display tissue-specific variation [2], with both T cells and antibodies recognizing mono- and disaccharide constituents of TACA. While most studies emphasize the cellular arm of the immune response in tumor rejection, the pathology observed from tumor-reactive antibodies can mirror autoimmune-mediated tissue damage [3], much like that observed for hyperacute rejection of xenotransplanted organs mediated by natural antibodies against the xeno-carbohydrate antigen Galα1-3Galβ1- 4GlcNAc-R (α-Gal) [4]. Augmentation of antibodies and T cells that are reactive with mono- and disaccharide constituents of TACA such as the Tn (GalNAcα-O-Ser/Thr), and Thomsen-Friedenreich (TF) (Galβ1-3GalNAcα/β-O-Ser/Thr) antigens are proposed to be of benefit for cancer survival, playing a role in immune surveillance and natural resistance to cancer [5]. However, the augmentation of immune responses to mono- and disaccharide TACA constituents raises the question of induction of reactivity to normal tissue because of the broad expression of small terminal groups on normal tissues.

To stimulate immune responses targeting TACAs we developed carbohydrate mimetic peptides (CMPs) that effectively promote tumor growth inhibition in mouse models of cancer [6-8]. We have shown that CMPs are broad-spectrum immunogens, inducing responses to multiple TACAs and therefore obviating the need for multivalent carbohydrate-based vaccines. Among CMPs are those that use aromatic-aromatic and hydrophobic interactions as critical chemical forces that modulate binding of the CMPs to anti-carbohydrate antibodies [9]. The tumor growth inhibition upon immunization of CMPs having a central motif of Tyr-Arg-Tyr or Trp-Arg-Tyr like CMP 106 with the sequence GGIYWRYDIYWRYDIYWRYD, the CMP 107 with the sequence GGIYYRYDIYYRYDIYYRYD and the CMP P10 with the sequence GVVWRYTAPVHLGDG are noted [6-8,10].

Because some TACAs expressed in mice are shared with humans, the natural expression of some of these fundamental glycan building blocks can parallel the human glycan expression patterns and therefore can account for host stromal interactions that can influence anti-glycan immune responses. Consequently, wild type mice can suffice to elucidate immune pathologies to ubiquitously expressed mono- and disaccharide elements of TACA. Before using these CMPs in human clinical trials it is necessary to demonstrate that immunization with CMPs are safe in that they do not induce immune pathology. CMPs react with human antibodies reactive with blood group antigens as well as gangliosides. The blood group mimicry of the CMPs was also suggested by their reactivity with the blood group reactive lectin *Griffonia simplicifolia* (GS-I), which recognizes α-galactosyl moieties. GS-I is a mixture of isolectins that bind to Galα1-3Gal and α-GalNAc terminal groups of disaccharides. GS-I is recognized as a surrogate marker to identify tumor expressed antigens reactive with α-Gal antibodies [11], and GS-I is of utility to interrogate α-GalNAc expression on both human and murine tissues [12]. The cross-reactivity of GS-I with murine and human cells and tissue can therefore be used to assess pathology in preclinical safety studies of immune responses to CMPs with the potential to cross-react with mono- and disaccharide moieties because the expression of GS-1 reactive antigens are presumed to be ubiquitously expressed in mice.

Our present studies demonstrate that activation of TACA reactive immune responses induced by CMP 107 and CMP 106 to a level sufficient to mediate therapeutic anti-tumor immunity *in vivo* can occur without the development of adverse immune pathology in mice as part of a preclinical safety study of CMPs. This low level immune response probably contributes to the lack of immune pathology associated with normal mouse tissue. The observation of a low level response to CMPs 106 and 107, albeit enough to mediate tumor growth inhibition, shifts the paradigm in thinking that a robust anti-tumor response is required for an effective therapy. These results clearly demonstrate striking context sensitivity in the immune recognition of endothelial cells expressing carbohydrate antigens, a subtlety that must be better understood for inducing immunity to tissue rejection antigens containing TACA to treat cancer and minimize complications involving immune pathology responses.

## 2. Results and Discussion

### 2.1. Tissue Distribution of GS-I Binding Is Restricted

The GS-I isotypes, GS-I-B4 and GS-I-A4, bind to group B and group A antigens, respectively, and exhibit strong binding to broadly expressed Galα1-2, Galα1-3 and Galα1-4 glycans [13]. Carbohydrate residues reactive with GS-I were previously shown to be present on the surface of highly malignant murine tumors but absent or expressed in much lower amounts on the surface of low malignant cells isolated from the same parent tumors [14]. To further study the expression pattern of GS-I-reactive glycans on tumors, we implanted murine 4T1 cells into mammary fat pads of a group of mice, and at day 35 post-transplant, mice were euthanized and sections of liver, lung and primary tumor mass were prepared and stained with GS-I. The 4T1 model closely resembles human breast cancer and is a rigorous model of advanced spontaneous metastatic disease, which metastasizes efficiently to lung, liver, bone, and brain after implantation into mammary fat pads [15].

**Figure 1.**
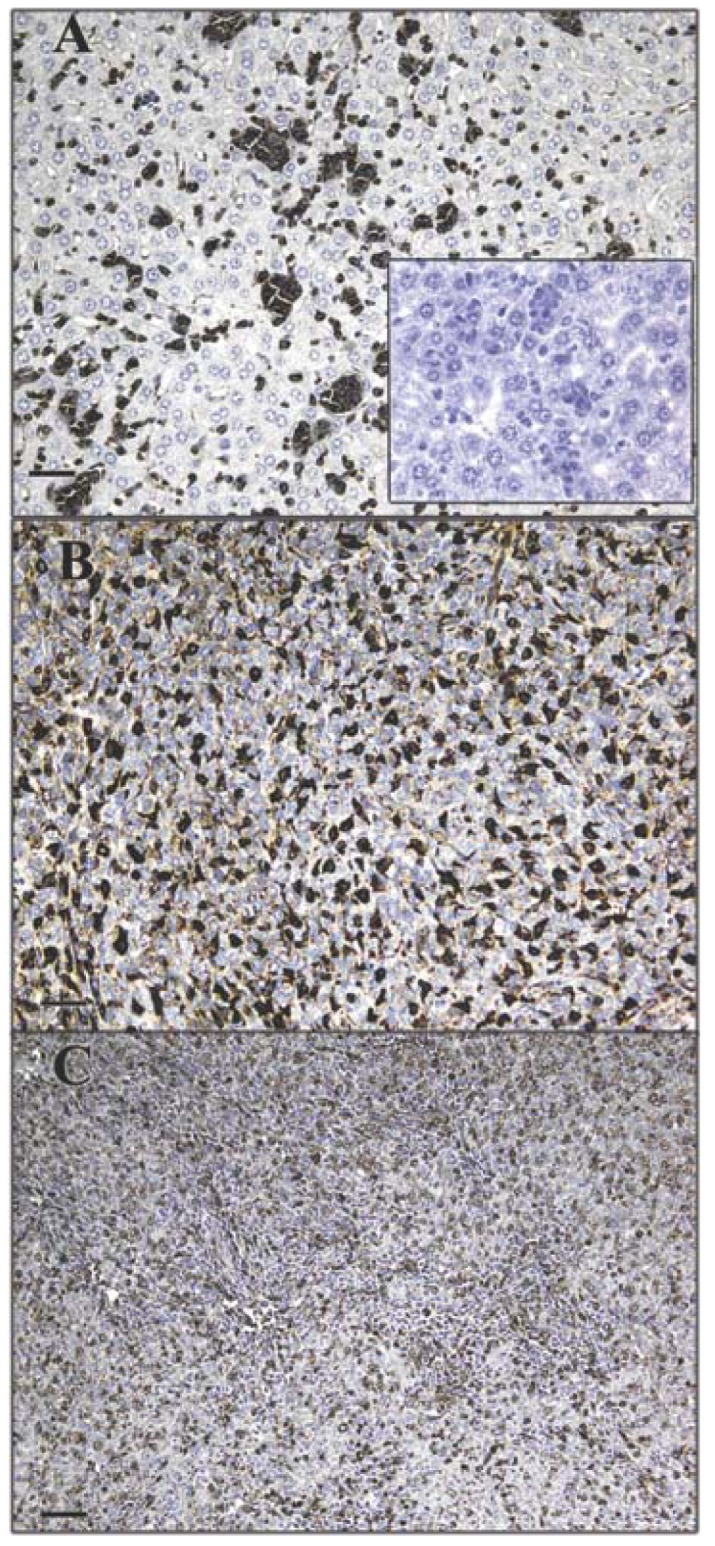
Expression pattern of GS-I binding to tumor cells. Murine 4T-1 tumor transplants were labeled with 2.5 μg/mL GS-I. Metastatic tumor cells in the liver (**A**) and lung (**B**) demonstrate increased GS-I binding compared to cells within the primary tumor (**C**). 200× magnification, bars equal 40 μm. No staining is observed in sections stained with biotin alone (**inset, A**).

**Figure 2.**
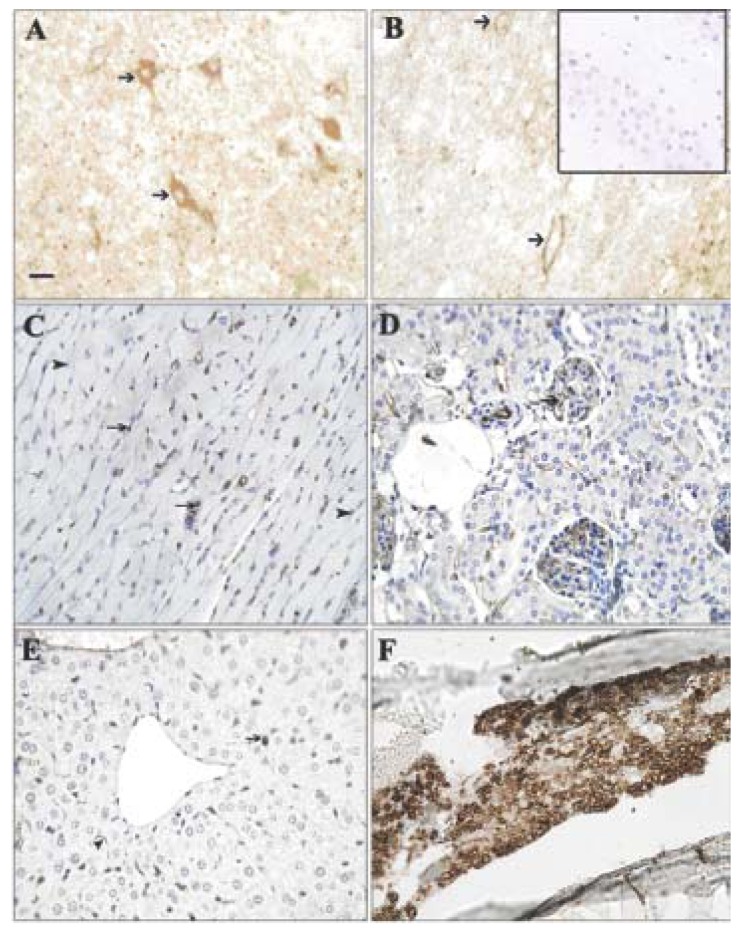
Expression pattern of GS-I binding to normal cells. Tissues from a naïve control mouse were labeled with GS-I. (**A**) Brain: Neurons (arrows) were labeled in a cytoplasmic and membranous fashion; (**B**) Spinal cord: Neurons were labeled in a membranous pattern; (**C**) Heart: Endothelial cells in the interstitium and in small arteries (arrows) were labeled. Myofibers (arrowheads) were not stained; (**D,E**) Kidney, Liver: Endothelial cells (arrows) bound GS-I weakly to moderately. Hepatocytes and renal tubular and glomerular epithelial cells did not bind GS-I; (**F**) Bone marrow: Hematopoietic cells bound GS-I strongly in a membranous and cytoplasmic pattern; (**A,B,C**) 200× magnification, bar equals 40 μm. (**D,E,F**) 400× magnification, bar equals 20 μm. No staining was observed in sections stained with biotin alone (**inset, B**).

Tumor cells in liver and lung metastases as well as the primary tumor were stained with GS-I, and staining in sections of metastatic lung and liver lesions were more intense than staining in the primary tumor (Figure 1). Lung metastases were detected as early as day 14 after transplantation in all mice tested, while liver metastases were detected between day 28 and day 35 after transplantation. In normal tissues other than hematopoietic cells at the same lectin concentration (2.5 μg/mL GS-I), GS-I staining was limited to endothelial cells and neurons (Figure 2) and was much less intense than the staining in either primary tumor sections or metastatic tumors. These results suggest that tumor cells on both primary 4T1 tumors and their metastases are enriched for GS-I binding sites compared with normal tissues. Our findings are consistent with previous observations that GS-I binds to endothelial cells and neurons of rodents and other animals [16,17]. GS-I reactive α-Gal epitopes on endothelial cells are presumably readily accessible to the immune system, and antibody recognition of these epitopes could lead to complement fixation and massive endothelial damage, as occurs in rejection of xenotransplants.

### 2.2. Similar Cell Expressed Epitopes Are Reactive with GS-I and Antibodies

**Figure 3.**
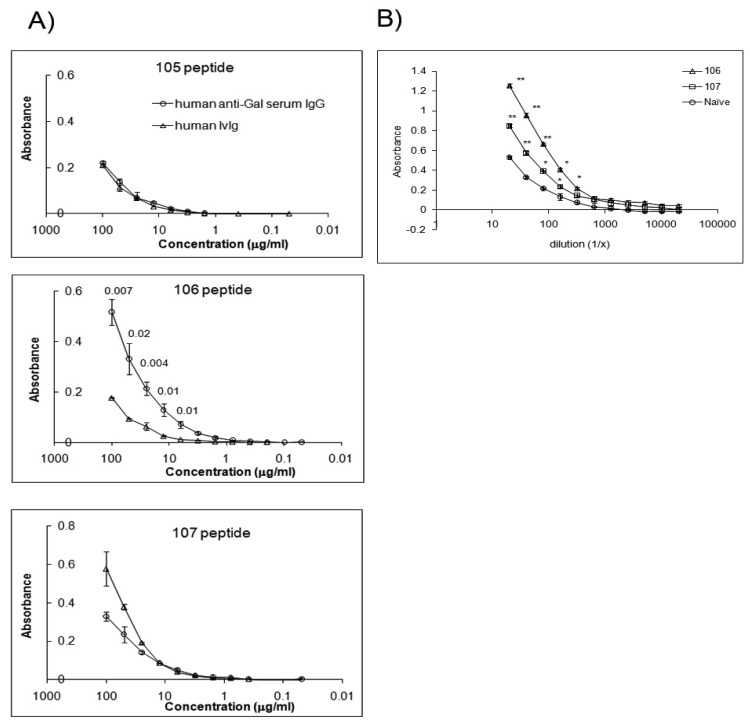
Anti-terminal Gal antibodies cross-react with CMPs and reactivity is increased after CMP immunization. Human anti-Gal antibodies bind to CMP 106 and CMP 107 and immunization with both CMPs induced IgM antibodies reactive with terminal alpha Gal. (**A**) ELISA plates were coated with the indicated peptides and reactivity of human serum enriched for anti-Gal fraction was detected. Human IVIg was used as negative control. Anti-Gal antibodies displayed distinct reactivity to CMP 106. Means (±SD) of triplicates in a single assay are shown. Student's t-test was used for statistical comparisons between anti-Gal serum and the control. P values for each individual concentration point with statistically significant differences are demonstrated; (**B**) Groups of 5 mice were immunized with CMP 106 and CMP 107. Serum was collected and pooled from 5 mice after the third immunization, and reactivity of the pooled sera was measured against Galα1,3GalB-PAA. Means (±SD) are estimated based on three replications. * and ** show significance at *P* < 0.05 and *P* < 0.01, respectively, as compared with naïve serum by Student's t-test.

The α-Gal epitope is considered a widely expressed self-antigen on murine tissue reactive with GS-I and anti-Gal antibodies. CMP 107 competes with antigen for GS-I binding, indicating that CMP 107 mimics GS-I-reactive carbohydrate chains [7]. Anti-α-Gal IgG antibodies enriched from human serum bind to 106 and not to 105 and 107 CMPs (Figure 3A). Both CMP 106 and CMP 107 induce serum antibodies in mice that react with structures containing terminal α-Gal (Figure 3B). The data suggest that anti-terminal Gal antibodies exist naturally (Figure 3B), and that reactivity is increased after immunization.

**Figure 4.**
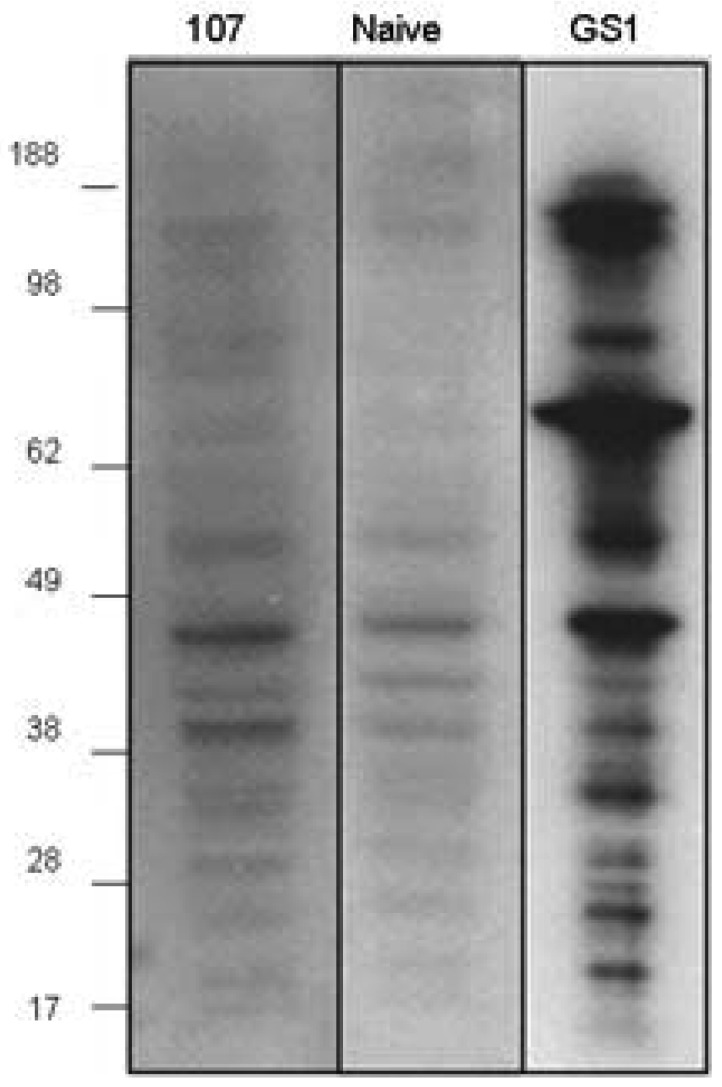
Serum IgM antibodies and GS-I binding to 4T1 cell lysate. Whole-cell lysate from 4T1 cell line was prepared. Mice (5/group) were pre-bled and immunized with peptide 107 on days 0 and 14. Sera were collected 7 days after the peptide boost and pooled for each group. Western blot analysis: Sera were diluted 1:1000 for the western. Anti mouse IgM used as secondary antibody. Binding with biotin-conjugated GS-I was followed by streptavidin-HRP.

The proapoptotic activity of CMP 107-induced antibodies to 4T1 cells parallels apoptotic activity observed upon GS-I binding of these cells, suggesting that apoptotic induction is via CMP 107-antibody recognition of GS-I ligands [7]. To examine the reactivity profile of GS-I and serum antibodies to similar ligands, blots of 4T1 cell extracts were probed. SDS-PAGE profiles of 4T1 cells probed with GS-I revealed several bands of varying molecular weight some of which were also identified, albeit weakly, by natural IgM antibodies in the naive (non-immunized) repertoire (Figure 4). Western blot analyses with CMP 107-induced serum antibodies displayed a pattern that paralleled the pattern of preimmune antibodies with intensified bands (Figure 4), suggesting that immunization with CMP 107 may amplify reactivities found in the preexisting naïve repertoire. Our results suggest that immunization with CMP 107 may amplify low-titer terminal-Gal reactive antibodies that are effectively mimicking GS-I reactivity for 4T1 cells.

**Figure 5.**
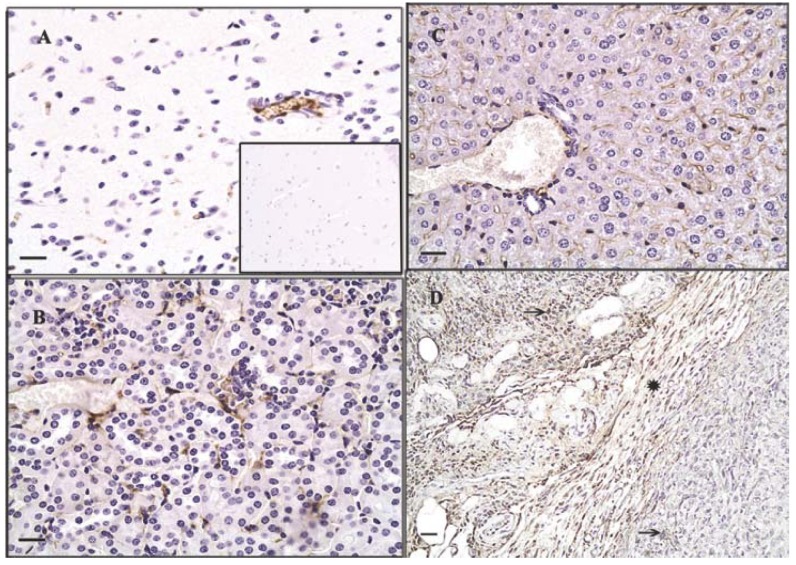
Deposition of immunoglobulin on normal tissue and tumor. Normal tissues (**A-C**) and tissue from a primary murine 4T-1 tumor (**D**) from non-immunized mice. 5 mice per group–one representative mouse- were immunostained with antibody against murine immunoglobulins. Endothelial cells in brain (**A**), kidney (**B**), liver (**C**), and primary tumor (**D**) were labeled with a distribution mirroring that of GS-I binding. IgG is bound to tumor cells (arrows) and stroma (star) in the primary tumor (**D**). 400× magnification, bars equal 20μm. No staining was observed in control sections labeled with antibody against goat immunoglobulin (**inset, A**).

**Figure 6.**
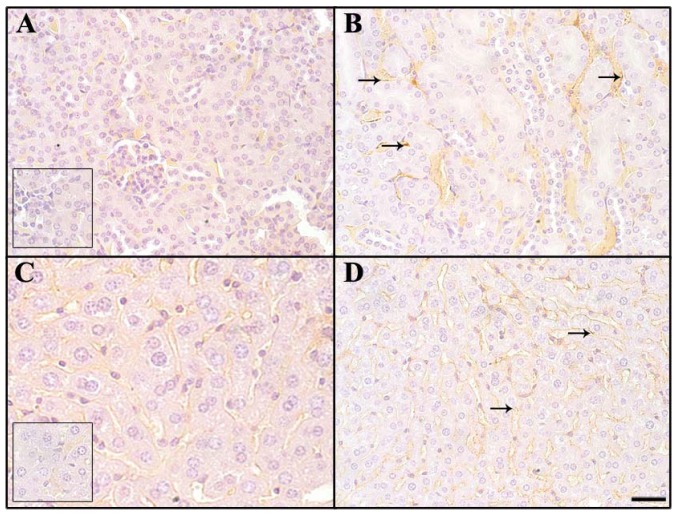
Comparison of IgG and IgM binding of tissues of non-immunized mice. Normal tissues were immunostained with antibody against naturally deposited murine IgG (**A,C**) and IgM (**B,D**). IgM staining is more intense, with a membranous and punctate pattern on endothelial cells (arrows). No staining is present in negative controls labeled with goat immunoglobulin (**inset A and inset C**). Kidney (**A,B**) and liver (**C,D**), 400× magnification, bar equals 40 μm.

To define further the tissue-reactive properties of natural antibodies, staining was performed to detect if immunoglobulin deposition occurred on normal tissue of non-immunized mouse control tissues. As with GS-I staining, deposition of immunoglobulin isotypes was detected on endothelial cells in the blood vessels of the kidney, liver, brain, and heart of each immunized mouse. Antibody deposition was not present outside the vasculature in these organs (Figures 5 A-C). In contrast, in tumor tissues, immunoglobulin deposits were detected on tumor cells and surrounding stroma (Figure 5D). In order to determine the relative contribution of IgG and IgM staining, we labeled tissues of naive mice separately with IgM or IgG. Staining with IgM was much more prominent than that of IgG (Figure 6). The staining distribution was similar to that obtained for total immunoglobulin, and was primarily punctate and membranous, suggesting aggregates on the endothelial cell surface (Figure 6). These data suggest that circulating preimmune antibodies, mostly of the IgM class display a tissue distribution and reactivity similar to that of GS-I, and that they might be amplified upon CMP immunization.

### 2.3. Immunization with CMPs Does Not Lead to Immunopathology

**Figure 7.**
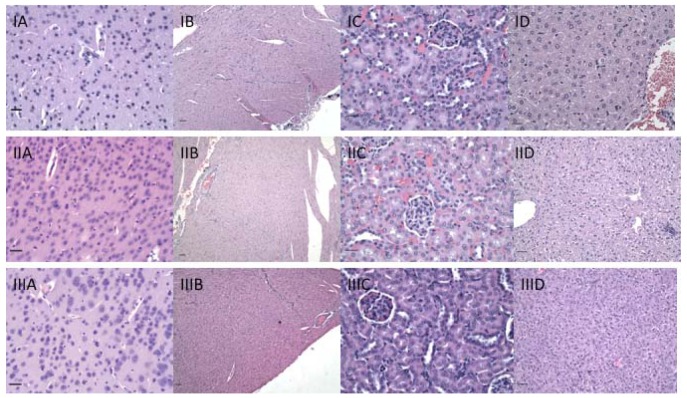
H&E staining of representative organs from immunized animals exhibiting no pathologic changes. I-CMP 106; II-CMP107; III-Control. **A**-cerebrum, 400×, bar equals 20 μm; **B**-heart, 100×, bar equals 100 μm; **C**-kidney, 200×, bar equals 50 μm; **D**-liver, 200×, bar equals 50 μm.

Because immunization with CMP 106 and CMP 107 induces anti-tumor immune responses, H&E stained sections of organs including the liver, kidney, heart, lungs, intestines, stomach, lymph nodes, spleen, brain, spinal cord, and eyes were examined to determine whether significant immunopathology was observed after immunization with either CMP 106 or 107 (Figure 7). No significant cellular infiltrates were identified in any organ, including the brain and spinal cord, from any animal in the short-term treatment groups, and there was no evidence of necrosis in these sections.

**Figure 8.**
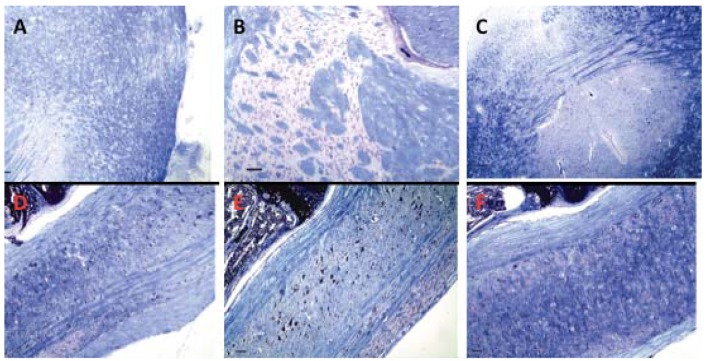
No evidence of demyelination is observed in brain or spinal cord of treated animals. CMP 106 (**A,D**); CMP 107 (**B,E**); Control (**C,F**). Luxol fast blue, 100× magnification, bar equals 100 μm.

Because alpha-Gal antigen is highly expressed in neural tissues (Figure 2), brain and spinal cord could be affected by immunopathology caused by formation of antibodies against this antigen. To rule out subtle changes in myelination we examined serial brain and spinal cord sections stained with Luxol fast blue for myelin. There was no difference in myelination between immunized and control animals (Figure 8). In animals immunized at various intervals for one year, mild, age-related changes were consistent with those in un-immunized, similarly aged mice.

Previous studies demonstrated that a significant lymphoid infiltrate was associated with regressing tumors upon CMP 106 immunization [8]. CMP 106 is hypothesized to mimic functionally GlcNAc expressed on glycopeptides associated with MHC Class I, which is recognized by T cells [8]. In contrast, we noted that cellular infiltrates were not found in normal tissues of animals immunized with CMP 106, further indicating that infiltrating T cells induced by CMP 106 are tumor-specific. Collectively these data demonstrate that although the titers of tumor-reactive antibodies induced in the CMP 106- and 107-immunized animals would be sufficient to cause tumor growth inhibition [6,7], histological analyses indicate no evidence of immunopathology in normal tissues.

Because low levels of antibodies able to react with most normal tissues can be identified in normal donors, there is concern that as the level of these antibodies increases due to immunization with tumor antigens, autoimmunity may develop [18]. The best-known example of carbohydrate targeting tissue damage is the natural antibody response in humans that is directed against Galα1-3Galβ1-4GlcNAc-R (α-Gal epitope). The α-Gal epitope is expressed on glycoproteins and glycolipids of non-primate mammals and is a major barrier in porcine-to-human xenotransplantation. Xenotransplant rejection is in large part due to destruction of organ endothelium by α-Gal-reactive antibodies that mediate complement activation as a mechanism of tissue damage. If TACA-directed antibodies could break the tolerance and induce antitumor responses in therapeutic or immune surveillance context comparable to the anti-xenotransplant ones, they may also mediate tissue damage.

Carbohydrates are accepted as clinically relevant antigens and the respective vaccines developed against infectious diseases might translate to cancer immunotherapy. The potential impact of TACA-directed vaccines is demonstrated in clinical trials where patient survival significantly correlates with carbohydrate-reactive IgM levels [19]. Such results suggest that TACA-targeting vaccines might have a beneficial effect on the course of malignant disease and TACA-induced responses could augment naturally occurring carbohydrate-reactive IgM antibodies that trigger the apoptosis of tumor cells [20,21]. A unique advantage in targeting TACAs is that multiple proteins and lipids on cancer cells can be modified with the same carbohydrate structure. Thus, targeting TACAs has the potential to broaden the spectrum of antigens recognized by the immune response, thereby lowering the risk of developing resistant tumors due to the loss of a given protein antigen.

CMPs of tumor antigen epitopes represent a potential novel vaccine approach to induce a tumor-specific humoral and cellular response and a strategy for inducing more robust immune responses to TACAs. Protein surrogates of TACAs are T-cell–dependent antigens and therefore immunization with these surrogates is predicted to facilitate cellular responses. CMPs have been described that induce antibodies against GD2 [22,23], GD3 [24], sialylated Lewis a/x [25], and LeY [6]. Although the characterization of CMPs is at present limited to preclinical studies, clinical characterizations of anti-idiotypic antibodies that mimic the GD3 ganglioside antigen [26] and GD2 [27] have been described. In addition, unlike carbohydrate antigens and carbohydrate-conjugate vaccines, we have shown that CMPs prime B- and T-cells for subsequent memory of carbohydrate antigens, facilitating long-term surveillance through recall of carbohydrate immune responses [28].

Due to flexibility and degenerate recognition, CMP 106 and CMP 107 functionally induce antibodies reactive with diverse TACAs. This feature obviates the need for developing a multivalent carbohydrate vaccine to induce broad-spectrum antibodies. As CMP 106 and CMP 107 are functional mimics of a broad spectrum of TACA, it might be argued that they would induce antibodies that would lead to tissue damage. To test this hypothesis we examined the tissue distribution pattern of terminal Gal reactive with GS-I and anti-CMP antibodies. We observed that GS-I-reactive ligands are present in neurons, endothelial cells, and hematopoietic cells of the bone marrow, and that natural antibodies, primarily IgM, bind to similar epitopes. We observed that CMP 107 binds to GS-I and, in mice, immunization with CMP 107 seems to boost reactivities in the preimmune repertoire of antibodies that bind to 4T1 cells. There was no evidence of inflammation, or necrosis, in any examined organ, and no significant differences were noted between control animals and vaccinated animals for either CMP. It is striking that antibody recognition of and binding to these antigens, especially on endothelial cells, does not seem to lead to the expected complement fixation and endothelial damage. This may be related to the polymeric form of the IgM (pentameric vs hexameric) as well as to the capacity of serum and cell membrane associated complement inhibitors. The absence of immunopathologies is also notable in neural tissues where the GS-I reactive antigen is most strongly expressed. No significant changes in myelin or in the degree of myelination were noted.

Antibodies recognizing Tumor Associated Antigens (TAAs) can lead to tumor-targeted immunopathology and tumor destruction; in this sense, the humoral response to TAAs resembles that of other immune responses to self-antigens. Collectively, the results of this preclinical study demonstrate that repeated injections of CMP 106 and CMP 107 do not lead to immune-mediated injury. These results are in concordance with reports from clinical trials using other types of cancer vaccines against these TACAs [29-33]. The underlying reasons for the absence of apparent immunopathology upon immunization with our xenoantigens are of interest. One definition of tolerance is the failure to mount a destructive immune response. By this definition, mice should be tolerant to GS-I reactive carbohydrates such as α-Gal despite generating specific antisera, because they do not appear to injure their own endothelial cells that express the antigen. Alternatively, tolerance has also been defined as unresponsiveness to an (otherwise) immunogenic antigen. By this definition, tolerance is not induced because an immune response is detected. Consequently, it is unclear why the activation of Gal reactive antibodies did not lead to pathology.

It is possible that the levels or patterns of expression of these TACA molecules on the surface of tumor cells differ significantly from that on normal cells. The increased level of GS-I binding we observed on tumor cells demonstrates that GS-I reactive antigens are up-regulated on tumor cells compared to normal cells. We demonstrated that immunoglobulin in naïve animals is deposited in the endothelium of several organs and in tumor cells, mirroring the binding of GS-I, and CMP immunization only amplifies moderately this reactivity. The recognition depends also on a threshold of avidity defined by the epitope's expression levels. Thus, fine specificity and quantitative thresholds are among a number of potential mechanisms that render the immune system tolerant to immunotherapy approaches that formally target self-epitopes. The enhancement of antibodies reactive with endothelial cells may be a partial explanation for the observed decreases in metastases to the liver of 4T1 cells following CMP immunization as endothelial cell to tumor contact is considered important for metastases [7].

Antibodies induced by CMPs are thought to have low affinities for TACAs. Thus, preferential targeting of tumor cells may be due in part to over-expression of TACAs on tumor cells, which compensates for the low affinity of the carbohydrate cross-reactive antibodies [34] and potential immunopathology due to minimization of destruction of normal tissue. Other features of the cell surface such as the three-dimensional arrangement of carbohydrate residues and characteristics of the protein such as size and valence may affect the expression patterns of TACAs on the cell surface and play critical roles in specific interactions on cell surfaces [11]. Multivalent binding by IgM may be a threshold-dependent phenomenon targeting only carbohydrate epitopes at high density.

A condition in which an organ transplant appears to flourish in the face of humoral immunity directed against it is called “accommodation” [35]. It is speculated that this condition could be important not only for overcoming humoral barriers to allotransplantation but also the more daunting barriers to xenotransplantation [35]. The “graft accommodation paradigm”, though, is useful because it stresses the importance of the high avidity site (in the case of transplantation–the graft, in our case the tumor) as a filter of the antibodies. In the case of grafts this notion is used to alert to apparently antibody negative cases that may be due to the adsorption of the circulating antibodies in the graft. The same notion here leads to a very different hypothesis–tumor-bearing individuals may be less at risk of autoimmunity than non-tumor bearing because the tumor acts as a sink of the antibodies produced. Yet our experiments were in clinically healthy mice. However, this effect might be related to the clustering or density of antigen and the avidity of the antibody. In the case of tumor bearing animals the deposition of antibodies as a sink means that the antibodies in themselves are not biologically relevant or that need support in the context of the tumor cell surface having complement to affect antibody mediated mechanisms or help from natural killer cells which work in concert with deposited antibodies.

**Figure 9.**
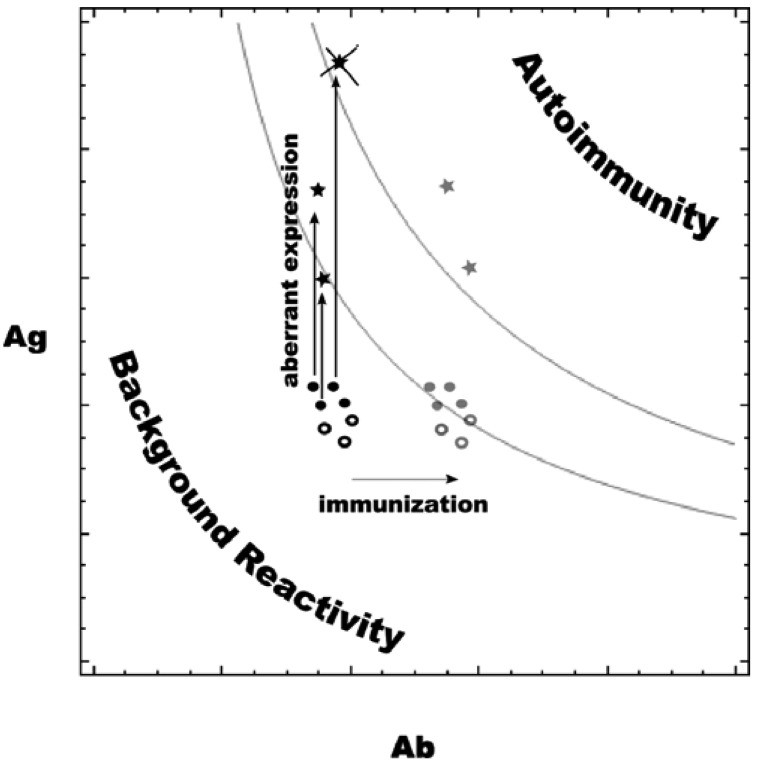
A model of the relationship between self/tumor-associated antigen densities (Ag) and natural antibody concentrations (Ab).

It is known that TACAs tend to cluster on the surface of tumor cells [29], and CMPs are designed to mimic that pattern. The increased expression of tumor-associated antigens may act in concert with the moderate increase in carbohydrate reactive antibodies induced by CMP immunization (Figure 9). The autoimmune pathology (and tolerance, respectively) at a given affinity level should depend on the concentration of the respective Ag–Ab complexes, which in its turn depends on Ag and Ab concentrations. Below certain values the concentrations of complexes Ag–Ab (formed by Ag and Ab concentrations below and to the left of the upper grey curve) are innocuous while the normal concentrations of Ag and Ab are probably such that Ag–Ab complexes are even less (below and to the left of the lower grey curve). The presented curves are derived as the places, for which:

$$\overset{1}{\;}/\underset{2}{\;}.\left[Ag+Ab+kd-\sqrt{-4.Ag.Ab+{\left(Ag+Ab+Kd\right)}^{2}}\right]=\text{const}$$

where Kd is the dissociation constant. This is actually the solution for [Ag–Ab] of the equation of a simple equilibrium binding based on the initial Ag and Ab concentrations. Simplified as it is, this concept helps map the quantitative changes during neoplastic transformation and tumor-associated antigen immunization. Self-antigens and the respective natural antibody concentrations define points on this map. Immunization with a self-antigen based vaccine that elicits moderately increased Ab concentrations is represented by a shift of the symbols to the right (grey symbols) if the respective Ag concentrations remain unchanged. At the same time, some of the tumor-associated self-antigens (filled black circles) are expressed at a higher level on the tumor (black star symbols) than on normal tissues (black round symbols). When this aberrant expression is high enough to trigger immune activity, the tumor will probably undergo immunoediting, and these epitopes will be lost (crossed out star symbol). For some of the others, the effect is such that only after immunization is the Ab concentration sufficient to produce an effective Ag–Ab concentration (grey star symbols). The ensuing autoimmune reactivity is directed toward the tumor. The expression on normal cells of tumor-associated (filled circles) and other (empty circles) antigens targeted by the vaccine remain lower, and the same shift in the antibody concentration only brings them in the safe zone below the autoimmunity threshold (respective grey symbols). These hypothetical quantitative relations explain the results reported in the presented study. This model also draws attention to the fact that it is easier to differentiate between normal and tumor tissue at lower affinities since at high affinity even small fluctuations in the antibody concentration can induce autoimmune phenomena in healthy tissues. Usually, autoimmune disease is a consequence, not of polyreactive IgM natural antibodies but of highly specific IgG antibodies directed against antigens on erythrocytes or thrombocytes. It would be interesting to establish the proportion of pentameric and hexameric IgM induced by CMP-based vaccines under different immunization protocols. In the case in which complement activation is inhibited by tumor cells, the formation of C3b fragments in association with IgM may be enough to trigger complement-dependent cellular cytotoxicity (CDCC) [36], which depends on Natural Killer cell sensitivity of the targets and provides another aspect of specificity. Finally, the accessibility of tumor antigens by IgM antibodies may be different than that of the normal tissues due to the decreased capacity of pentameric and hexameric IgM to leave the vascular bed combined with the high leakiness of the tumor vessels.

In particular, we confirmed the endothelial-specific GS-I lectin staining pattern and showed that normal serum antibodies display a similar staining pattern, which increases after CMP immunization. Antibody-mediated tissue damage in autoimmunity is usually only considered to occur when the autoantibody recognizes accessible antigens. In other words, the antigen should be either free in the extracellular fluid or expressed upon the cell surface, for the circulating antibodies to bind it and form immune complexes. Our results support a model where shared antigens expressed on normal tissue and tumor cells can be accessible to the immune system. This suggests that the expression of these antigens is immunologically ignored not through repertoire holes but by a quantitative threshold mechanism. Thus, natural autoreactivity in the antibody repertoire are tuned to a range of binding parameters that serve as first line of defense and surveillance but avoid autoaggression. This repertoire may prove to have a margin of further activation resiliently controlled (limited above) to yield functional tolerance. Thus, as our results further indicate, the endothelium is capable of presenting antigen to antibodies, and this presentation neither results in anergy or deletion of carbohydrate specific B cells, nor prevents the generation of a functional immune response against tumor cells.

## 3. Experimental Section

### 3.1. CMP Synthesis and Immunization

Animal studies were reviewed and approved by the Institutional Care and Use Committee of the University of Arkansas for Medical Sciences. CMP 106 with the sequence GGIYWRYDIYWR YDIYWRYD and CMP 107 with the sequence GGIYYRYDIYYRYDIYYRYD were synthesized as multiple antigen peptides (MAPs) (Bio-Synthesis inc., Lewisville, TX, USA) made by FMoc synthesis on poly-L-lysine groups resulting in the presentation of eight peptide clusters [6]. BALB/c mice were purchased from the Jackson Laboratory (Bar Harbor, ME, USA). Mice were randomly assigned to four groups (N = 5). Two groups were immunized three times at two-week intervals via subcutaneous injection with either 100 μg of Map 106 or Map 107 with 20 μg QS-21 admixed with 20 μg of keyhole limpet hemocyanin (KLH) in 100 μL of sterile PBS as adjuvant. One adjuvant control group received only 20 μg QS-21 and KLH, and a naïve control group was un-immunized. To determine the effects of long-term repeated exposures, a separate group of four mice was similarly immunized with 100 μg of MAP 106 six times during a one-year period (long-term treatment group).

### 3.2. Necropsy and Histopathology

Fourteen days after the last injection, animals were euthanized via overdose of CO_2_. Cardiac puncture was performed immediately post-mortem to obtain blood. A complete necropsy was performed, and organs were placed immediately into 10% neutral buffered formalin (NBF). Organ weights were obtained for liver, heart, left and right kidneys, spleen, and lung. The brain was fixed in situ in 10% NBF. Tissues were processed and embedded in paraffin and sectioned at 6μm. Sections were stained with hematoxylin and eosin for routine histologic evaluation. Sections of brain and spinal cord were stained using the Luxol-fast blue technique to identify myelin.

### 3.3. Western Blotting

Proteins (40 μg) were separated on gradient 4–12% SDS-PAGE (Invitrogen, Carlsbad, CA, USA) and transferred to polyvinylidene fluoride membranes (Invitrogen). The membranes were blocked with 3% BSA containing 0.01% Tween-20 overnight in Phosphate Buffer Saline (PBS; pH 7.6) at 4 °C and then incubated with serum (1:1000 dilution) or 2 μg/mL of biotinylated GS-I lectin in PBS containing 1% BSA and 0.01% Tween-20 for 2 hr at room temperature. After the membranes were washed, they were incubated for 1 hr at room temperature with peroxidase-conjugated streptavidin (0.25 μg/mL) or an anti-mouse IgM antibody (1:2000 dilution) in PBS containing 0.01% Tween-20. For visualization, an enhanced chemiluminescence-based detection system (Amersham ECL^™^, GE Healthcare Bio-sciences Corp., Piscataway, NJ, USA) was used and the membranes were exposed to X-ray film (Eastman Kodak Co; Rochester, NY, USA).

### 3.4. Lectin Histochemistry

Sections were deparaffinized, hydrated through xylenes and graded alcohol series, and washed in DPBS. Antigen retrieval with 0.1 M citrate buffer (pH 6.0) for 40 min at 100 °C was performed. Endogenous peroxidase activity was blocked by immersion in 0.3 % (w:v) hydrogen peroxide in absolute methanol for 10 min followed by DPBS wash. Nonspecific binding was blocked by incubating with DPBS containing 2% BSA at RT for 35 min. Sections were then incubated with 2.5 μg/mL GS-I-biotinylated for 1 hr at RT and washed in DPBS containing 0.1% Tween-20. Sections were then incubated with streptavidin horseradish peroxidase (Dako Cytomation, Carpinteria, CA, USA) for 30 min at RT, followed by incubation with DAB solution for 2 min at RT, then rinsed in distilled water. Slides were counterstained with hematoxylin for 30 s, cleared and mounted. Slides were stained using biotin only as a negative control.

### 3.5. Immunohistochemistry for Immunoglobulin Deposition

Sections were deparaffinized, hydrated through xylenes and graded alcohol series, and washed in DPBS. Antigen retrieval with 0.1% Trypsin (Mediatech Inc, Herndon, VA, USA) for 5 min at RT was performed. Endogenous peroxidase activity was blocked by immersion in 0.3% (w:v) hydrogen peroxide in absolute methanol (Dako Cytomation) for 10 min followed by DPBS wash. Nonspecific binding was blocked by incubating with DPBS containing 2% BSA at RT for 35 min. Sections were then incubated with a biotinylated mixture of anti-mouse IgG and anti-mouse IgM (Dako Cytomation) for 1 hr at RT and washed in DPBS containing 0.1% Tween 20, followed by incubation with streptavidin horseradish peroxidase (1:5000, Pierce, Rockford, IL, USA) for 30 min at RT. Sections were then incubated with DAB solution (Zymed Laboratories Inc, South San Francisco, CA, USA) for 2 min at RT, washed in distilled water. Slides were counterstained with hematoxylin for 30 s, cleared, and mounted. To determine relative reactivity of IgG and IgM, slides were separately stained with biotinylated anti-murine IgG (Sigma, St. Louis, MO, USA, 1:400) and –IgM (Vector, Burlingame, CA, USA, 1:400) antibodies. Slides were stained using a biotinylated anti-goat antibody and with secondary antibody alone as negative control.

### 3.6. Statistical Analyses

Student's t test was used to compare means. Differences were considered significant if P was <0.05. Significant P values are presented for each figure. All assays were repeated at least three times.

## 4. Conclusions

In summary, this study supports the development of CMPs for clinical testing. The ability of CMPs to induce antibodies reactive with multiple TACAs is relevant as heterogeneity of antigen expression in different cancers of the same type, as well as different cells of the same cancer, and heterogeneity of immune response in different patients makes it likely that maximal anticancer effect may not result from immunization against a single antigen. Consequently, immunizations with polyvalent vaccines containing several TACAs or immunization with CMPs that functionally emulate several TACAs are a viable strategy in vaccine development and are not expected to cause immunopathology. Our results suggest that these vaccines will be safe for long-term treatment, and larger preclinical safety studies are expected to verify these findings. Adding these vaccines to the treatment armamentarium may significantly improve outcomes for patients affected with breast cancer and other types of tumors.
